# Supplementary material for: Hybrid Models and Biological Model Reduction with PyDSTool
Source: PLoS Comput Biol. 2012 Aug 9;8(8):e1002628. doi: 10.1371/journal.pcbi.1002628 (PMC3415397; doi:10.1371/journal.pcbi.1002628)
Supplement: Text S4 — Complete source code for the PyDSTool package (version 0.88.120504). Includes API documentation and help files linking to web pages. This file is identical to the current public release on Sourceforge.net. (ZIP) [file pcbi.1002628.s004.zip › PyDSTool/html/PyDSTool.Toolbox.FSM.ObjFSM-class.html]

xml version="1.0" encoding="ascii"?


PyDSTool.Toolbox.FSM.ObjFSM


| Home | Trees | Indices | Help | PyDSTool |
| --- | --- | --- | --- | --- |

Package PyDSTool ::
Package Toolbox ::
Module FSM ::
Class ObjFSM

[hide private]

[frames] | no frames]

# Class ObjFSM

source code

```
object --+    
         |    
       FSM --+
             |
            ObjFSM
```

---

A subclass of FSM where input\_symbol may be any kind of object, even
an unhashable one. For each input\_symbol to process, the machine will try
a sequence of functions; the first to return True determines (action,
next\_state).


|  |  |
| --- | --- |
| Instance Methods | [hide private] |

|  |  |
| --- | --- |
| add\_transition(self, test, state, action, next\_state)  This adds a transition that associates: | source code |

|  |  |
| --- | --- |
| get\_transition(self, input\_symbol, state)  This returns (action, next state) given an input\_symbol and state. | source code |

**Inherited from `FSM`**:
`__init__`,
`add_transition_any`,
`add_transition_list`,
`process`,
`process_list`,
`reset`,
`set_default_transition`

**Inherited from `object`**:
`__delattr__`,
`__getattribute__`,
`__hash__`,
`__new__`,
`__reduce__`,
`__reduce_ex__`,
`__repr__`,
`__setattr__`,
`__str__`


|  |  |
| --- | --- |
| Properties | [hide private] |

**Inherited from `object`**:
`__class__`


|  |  |
| --- | --- |
| Method Details | [hide private] |

|  |  |
| --- | --- |
| add\_transition(self, test, state, action, next\_state) | source code |

```
This adds a transition that associates:

        (input_symbol, current_state) --> (action, next_state)

The action may be set to None in which case the process() method will
ignore the action and only set the next_state. The next_state may be
set to None in which case the current state will be unchanged.

You can also set transitions for a list of symbols by using
add_transition_list().
```

Overrides: FSM.add\_transition: *(inherited documentation)*

|  |  |
| --- | --- |
| get\_transition(self, input\_symbol, state) | source code |

```
This returns (action, next state) given an input_symbol and state.
This does not modify the FSM state, so calling this method has no side
effects. Normally you do not call this method directly. It is called by
process().

The sequence of steps to check for a defined transition goes from the
most specific to the least specific.

1. Check state_transitions[] that match exactly the tuple,
    (input_symbol, state)

2. Check state_transitions_any[] that match (state)
    In other words, match a specific state and ANY input_symbol.

3. Check if the default_transition is defined.
    This catches any input_symbol and any state.
    This is a handler for errors, undefined states, or defaults.

4. No transition was defined. If we get here then raise an exception.
```

Overrides: FSM.get\_transition: *(inherited documentation)*

  


| Home | Trees | Indices | Help | PyDSTool |
| --- | --- | --- | --- | --- |

|  |  |
| --- | --- |
| Generated by Epydoc 3.0.1 on Fri May 4 15:24:08 2012 | http://epydoc.sourceforge.net |
